# Pharmacological inhibition of cryptochrome and REV-ERB promotes DNA repair and cell cycle arrest in cisplatin-treated human cells

**DOI:** 10.1038/s41598-021-97603-x

**Published:** 2021-09-09

**Authors:** Nadeen Anabtawi, William Cvammen, Michael G. Kemp

**Affiliations:** Department of Pharmacology and Toxicology, Wright State University Boonshoft School of Medicine, Dayton, OH 45435 USA

**Keywords:** Cell biology, Cell growth, Circadian rhythms, Cancer, Cancer therapy, Biochemistry, DNA

## Abstract

Nucleotide excision repair (NER) and cell cycle checkpoints impact the ability of the anti-cancer drug cisplatin to inhibit cell proliferation and induce cell death. Genetic studies have shown that both NER and cell cycle progression are impacted by the circadian clock, which has emerged as a novel pharmacological target for the treatment of various disease states. In this study, cultured human cell lines were treated with combinations of cisplatin and the circadian clock modulating compounds KS15 and SR8278, which enhance circadian clock transcriptional output by inhibiting the activities of the cryptochrome and REV-ERB proteins, respectively. Treatment of cells with KS15 and SR8278 protected cells against the anti-proliferative effects of cisplatin and increased the expression of NER factor XPA and cell cycle regulators Wee1 and p21 at the mRNA and protein level. Correlated with these molecular changes, KS15 and SR8278 treatment resulted in fewer unrepaired cisplatin–DNA adducts in genomic DNA and a higher fraction of cells in the G1 phase of the cell cycle. Thus, the use of pharmacological agents targeting the circadian clock could be a novel approach to modulate the responses of normal and cancer cells to cisplatin chemotherapy regimens.

## Introduction

Cisplatin and other platinating agents are widely used in the treatment of different cancers due to their formation of adducts on DNA that interfere with DNA replication and induce cell death in rapidly proliferating cancer cells^1,2^. The nucleotide excision repair (NER) system plays an essential role in removing intra-strand cisplatin–DNA adducts from the genome^3,4^ and therefore limits the effectiveness of cisplatin chemotherapy regimens. Cell cycle checkpoints and other components of the cellular DNA damage response also impact cisplatin responses by allowing additional time for DNA repair prior to the entry of cells into S phase and mitosis^5,6^, during which time unrepaired DNA damage may be particularly problematic.

An additional system that has emerged as a regulator of the DNA damage response is the body’s circadian clock^7^, which governs many aspects of cellular, tissue, and organismal biochemistry and physiology with a periodicity of 24 h. At the molecular level, the circadian clock is composed of a transcription-translation feedback loop in which the CLOCK-BMAL1 heterodimeric protein complex binds to E-box elements in promoters of target clock-control genes (CCGs) to modulate gene expression^8^. Two target CCGs include cryptochrome (CRY) and period (PER), which ultimately heterodimerize and feedback to inhibit CLOCK-BMAL1 activity. A secondary loop is governed by retinoic acid receptor orphan receptor (ROR) and REV-ERB proteins that bind in a competitive manner to ROR elements in the BMAL1 promoter to influence BMAL1 expression^9^. It has been estimated that up to 40–50% of all genes are regulated by the circadian clock in at least one tissue^10^, and thus it is expected that some of these gene products likely influence cisplatin DNA damage responses. For example, genes encoding the rate-limiting NER factor XPA (xeroderma pigmentosum group A) and anti-mitotic protein kinase Wee1 are well-recognized transcriptional targets of the circadian clock^11,12^. Additional studies have found that the time of exposure during the 24 h day to cisplatin and other related DNA damaging agents like UV radiation impacts the efficiency of DNA repair and corresponding cell lethality^13–17^.

Genetic studies in cultured cells and mice have dominated experimental analyses of the circadian clock and its role in the pathophysiology of numerous disease states over the past 20 years^7,18,19^. However, recent drug discovery screens have identified compounds that bind to and regulate protein components of the circadian clock machinery^20,21^. These small molecules may therefore provide a new way to modulate cell and tissue biology under normal or pathological conditions and in response to external stressors, such as chemotherapy. The purpose of the current study was to examine whether circadian clock-modulating compounds can be used to alter the expression of genes relevant to cisplatin DNA damage responses and thus control the anti-proliferative effects of this common anti-cancer therapeutic drug in cultured cells in vitro.

## Results

### The CRY inhibitor KS15 and REV-ERB antagonist SR8278 promote cell proliferation in cisplatin-treated cells

**Figure 1:**
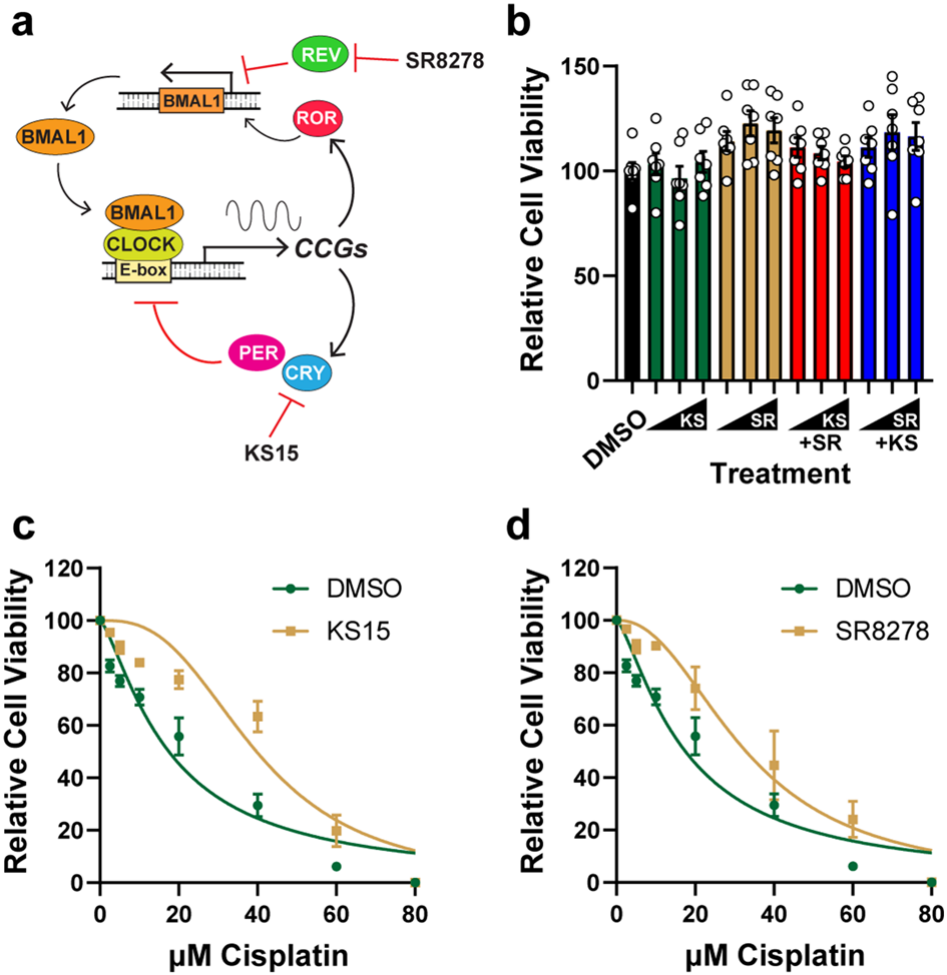
The cryptochrome inhibitor KS15 and REV-ERB antagonist SR8278 protect U2OS cells against cisplatin treatment. **(a)** Schematic of the circadian clock transcription-translation feedback loop. The CLOCK-BMAL1 complex binds E-boxes in the promoters of target clock-control genes (CCGs), including the period (PER) and cryptochrome (CRY) that feedback to inhibit CLOCK-BMAL1 activity. Expression of BMAL1 is regulated by the retinoic acid receptor-related orphan receptor (ROR) and REV-ERB (REV) proteins that competitively bind to ROR elements (RORE) in the BMAL1 promoter. The CRY inhibitor KS15 and REV-ERB antagonist SR8278 have been shown to impact target gene and promoter activities. **(b)** U2OS cells were treated for 2 days with vehicle (0.1% DMSO) or increasing concentrations (10, 20, 50 µM) of KS15 (KS) and SR8278 (SR) in the absence or presence of 10 µM of the other compound. MTT assays were performed to measure relative cell viability (average and SEM, n = 7). **(c)** U2OS cells were treated with DMSO or 50 µM KS15 and the indicated concentration of cisplatin for 2 days, and then MTT assays were performed to measure relative cell proliferation (average and SEM; n = 6). **(d)** U2OS cells were treated as in **(c) **except with 50 µM SR8278.

A schematic of the circadian clock transcription-translation feedback system is provided in Fig. 1A along with the two clock-modulating compounds (KS15 and SR8278) that were tested in this study due to their documented ability to alter circadian promoter activity and/or CCG expression^22–24^. CRY represses CLOCK-BMAL1 transcriptional activity, and the CRY inhibitor KS15 has been shown increase in the expression of CCGs such as Wee1 in cells in vitro^22,23^. REV-ERB, which negatively regulates BMAL1 expression by binding to its promoter, can be antagonized with SR8278^24^. Because the effectiveness of the anti-cancer drug cisplatin is impacted by cellular processes that are regulated by the circadian clock, such as DNA repair and cell cycle checkpoints^18,25^, the effect of KS15 and SR8278 on DNA repair, cell cycle progression, and cell viability were tested both alone and in various combinations.

U2OS cells were selected for this initial analysis because this cell line expresses circadian proteins and maintains circadian rhythmicity upon stimulation^26^. As shown in Fig. 1B, treatment of asynchronously growing U2OS cells with increasing concentrations of KS15 and SR8278 (10, 20, and 50 µM) did not dramatically influence cell proliferation either alone or in combination with a fixed concentration (10 µM) of the other compound. Cells were then co-treated with the clock drugs in combination with increasing concentrations of cisplatin. Interestingly, treatment of U2OS cells with 50 µM KS15 partially protected cells against the anti-proliferative effects of cisplatin (Fig. 1C). Similar results were observed with 50 µM SR8278 (Fig. 1D). Additional cell proliferation assays were carried out with various concentrations of KS15 and SR8278 alone and in combination, and IC_50_ values for cisplatin were calculated with the different treatment combinations. As shown in Table 1, the highest concentrations of KS15 and SR8278 led to statistically significant changes in IC_50_ values, which increased from 18 µM in vehicle-treated cells to approximately 40 µM in cells treated with the clock modulating compounds. Similar levels of protection were also observed in cells treated with a combination of lower concentrations of KS15 and SR8278.

**Table 1:** Summary of IC_50_ values for cisplatin-treated human cells.

Cell line	Treatment	Average IC_50_	SEM	p-value
U2OS	DMSO	18.4	2.15	–
	10 µM KS15	25.6	2.01	0.811
	20 µM KS15	29.9	3.50	0.290
	50 µM KS15	41.9	4.11	0.001
	10 µM SR8278	25.1	3.19	0.864
	20 µM SR8178	25.8	3.75	0.781
	50 µM SR8178	37.1	6.11	0.014
	10 µM SR + 10 µM KS	33.3	3.72	0.082
	10 µM SR + 20 µM KS	35.5	3.99	0.031
	10 µM SR + 50 µM KS	40.8	3.72	0.002
	10 µM KS + 20 µM SR	36.7	5.29	0.018
	10 µM KS + 50 µM SR	41.3	4.94	0.001
HaCaT	DMSO	3.2	0.18	–
	10 µM KS15	4.9	0.56	0.711
	20 µM KS15	4.9	0.85	0.723
	50 µM KS15	6.0	1.21	0.237
	10 µM SR8278	4.5	0.49	0.865
	10 µM SR + 10 µM KS	5.8	1.07	0.306
	10 µM SR + 20 µM KS	6.6	1.44	0.118
	10 µM SR + 50 µM KS	7.4	1.34	0.042
A549	DMSO	9.4	1.55	–
	10 µM KS15	9.9	1.84	0.999
	20 µM KS15	12.5	1.06	0.661
	50 µM KS15	12.8	2.39	0.574
	10 µM SR8278	8.6	1.04	0.999
	10 µM SR + 10 µM KS	12.6	0.91	0.633
	10 µM SR + 20 µM KS	15.3	2.52	0.121
	10 µM SR + 50 µM KS	17.3	1.39	0.023

**Figure 2:**
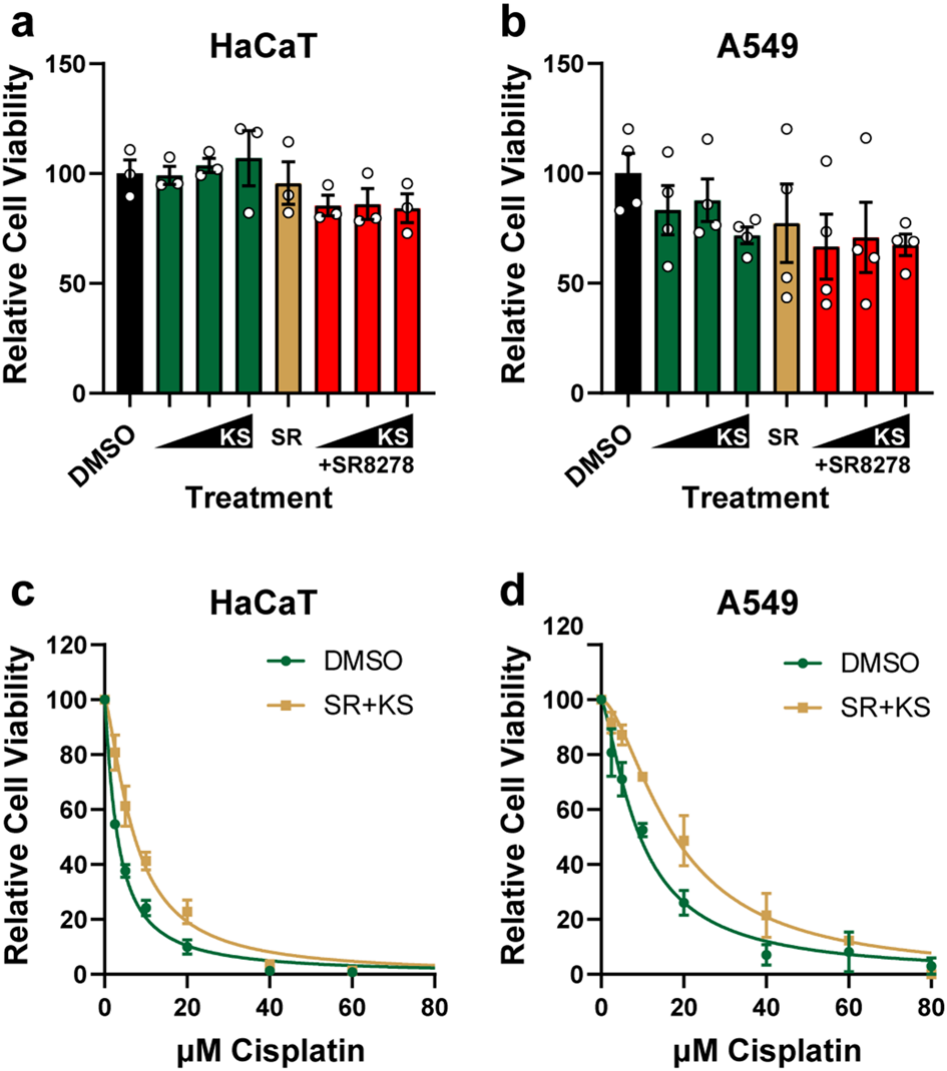
KS15 and SR8278 protect HaCaT and A549 cells against cisplatin treatment. **(a)** MTT assays were performed in HaCaT cells treated with KS15 and SR8278 (average and SEM, n = 3). **(b)** HaCaT cells were treated with DMSO or a combination of 50 µM KS15 and 10 µM SR8278 along with the indicated concentrations of cisplatin. MTT assays were performed two days later (average and SEM, n = 3). **(c)** A549 cells were treated as in (a), and MTT assays were performed (average and SEM, n = 4). **(d)** MTT assays were performed in A549 cells were treated with cisplatin and DMSO or the combination of 50 µM K15 and 10 µM SR8278 (average and SEM, n = 4).

Non-linear regression was used to calculate IC_50_ values for U2OS, HaCaT, and A549 cells treated with cisplatin and the indicated concentrations of KS15 and SR8278 as shown in Figs. 1 and 2. One-way ANOVAs were used to identify KS15 and SR8278 treatment conditions that significantly impacted IC_50_ values in comparison to the vehicle (DMSO)-treated control.

To confirm these results in additional cell lines, HaCaT keratinocytes, which also have a functional circadian clock^27^, were treated with KS15 alone and in combination with SR8278. As shown in Fig. 2A, the compounds alone had no significant effect on cell proliferation. However, when HaCaT cells were treated with 50 µM KS15 in combination with 10 µM SR8278 in the presence of increasing concentrations of cisplatin, they displayed significantly higher levels of cell proliferation than vehicle-treated cells (Fig. 2B, Table 1). Experiments were then repeated in A549 lung carcinoma cells, which displayed little sensitivity to the clock drugs alone (Fig. 2C) but were protected against cisplatin by co-treatment with KS15 and SR8278 (Fig. 2D, Table 1). Thus, we conclude from these studies that the clock modulating compounds KS15 and SR8278 can be used to counteract the anti-proliferative effects of the cancer chemotherapy drug cisplatin in cultured human cells in vitro.

### KS15 and SR8278 treatment increases the expression of the CCGs XPA and Wee1

**Figure 3:**
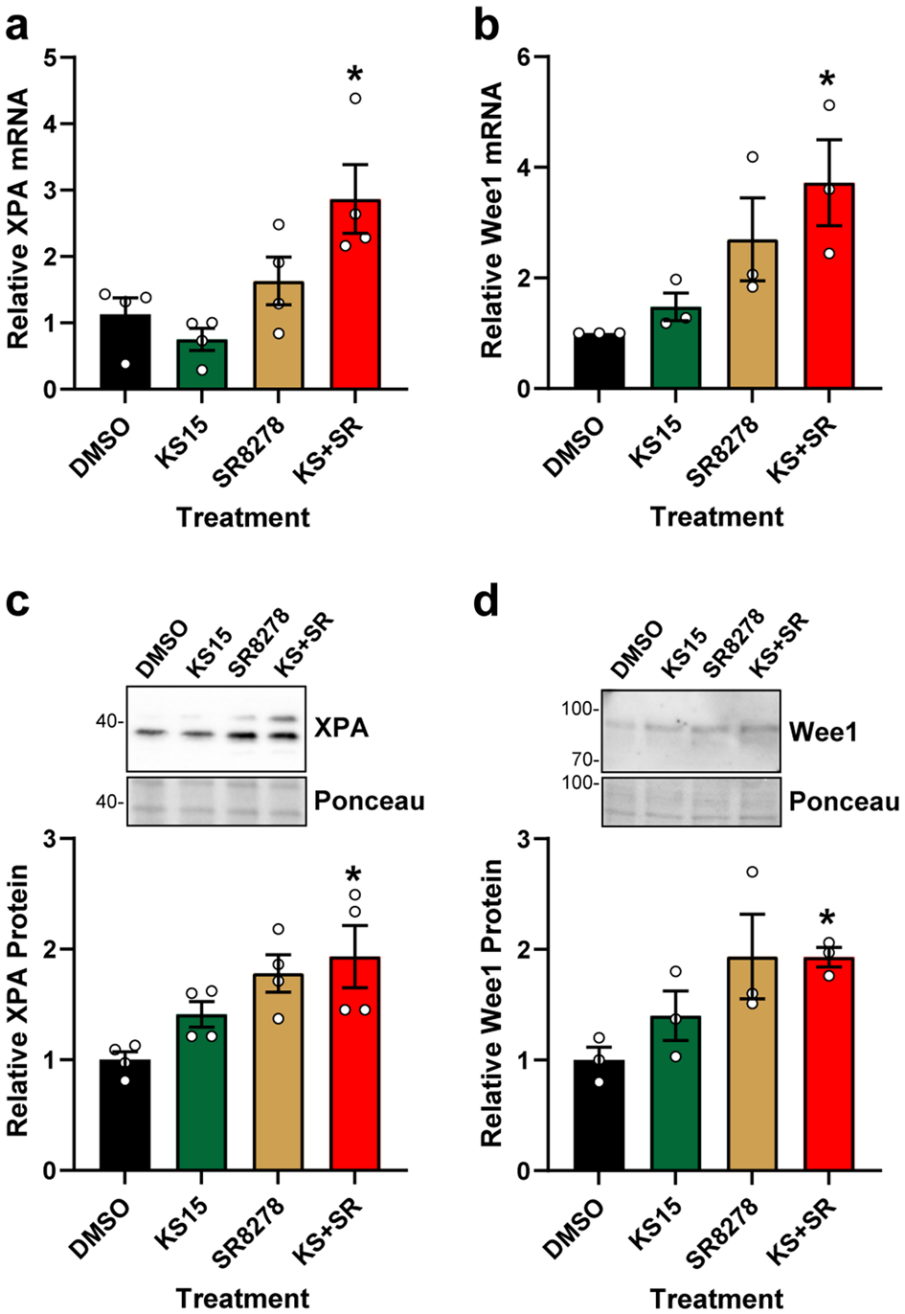
KS15 and SR8278 treatment increase XPA and Wee1 expression in U2OS cells. **(a)** U2OS cells were treated with DMSO vehicle, 20 µM KS15, 10 µM SR8278, or the combination of KS15 and SR8278 (KS + SR) for 24 h. RNA was purified and analyzed for XPA mRNA expression by RT-qPCR (average and SEM, n = 4). **(b)** Wee1 mRNA was analyzed as in **(a)**. **(c)** Cell lysates from cells treated as in **(a)** were analyzed for XPA protein by immunoblotting (average and SEM, n = 4). **(d)** Wee1 protein expression was analyzed as in **(c)**. Full-length blots are presented in Supplementary Figure S1. The asterisks indicate differences in mRNA protein expression in comparison to DMSO-treated control cells (p < 0.05; one-way ANOVA).

Cellular sensitivity to cisplatin is impacted by many factors, including DNA repair and cell cycle phase^3,28,29^. Because the NER factor XPA is regulated at the transcriptional level by the circadian clock machinery^11,14^, XPA mRNA expression was measured by RT-qPCR in U2OS treated with KS15 and SR278 alone and in combination. As shown in Fig. 3A, treatment with the combination of KS15 and SR8278 increased XPA expression by approximately 2.9-fold. Expression of Wee1, another well-known target of the circadian clock^12^, was also increased by KS15 + SR8278 by 3.7-fold (Fig. 3B). To determine if the increased mRNA levels are correlated with changes at the protein level, western blotting was performed using cell lysates from U2OS cells treated with the clock compounds. As shown in Fig. 3C,D, protein levels of both XPA and Wee1 were increased by nearly two-fold in cells treated with the combination of KS15 and SR8278.

### KS15 and SR8278 promote cisplatin–DNA adduct removal

**Figure 4:**
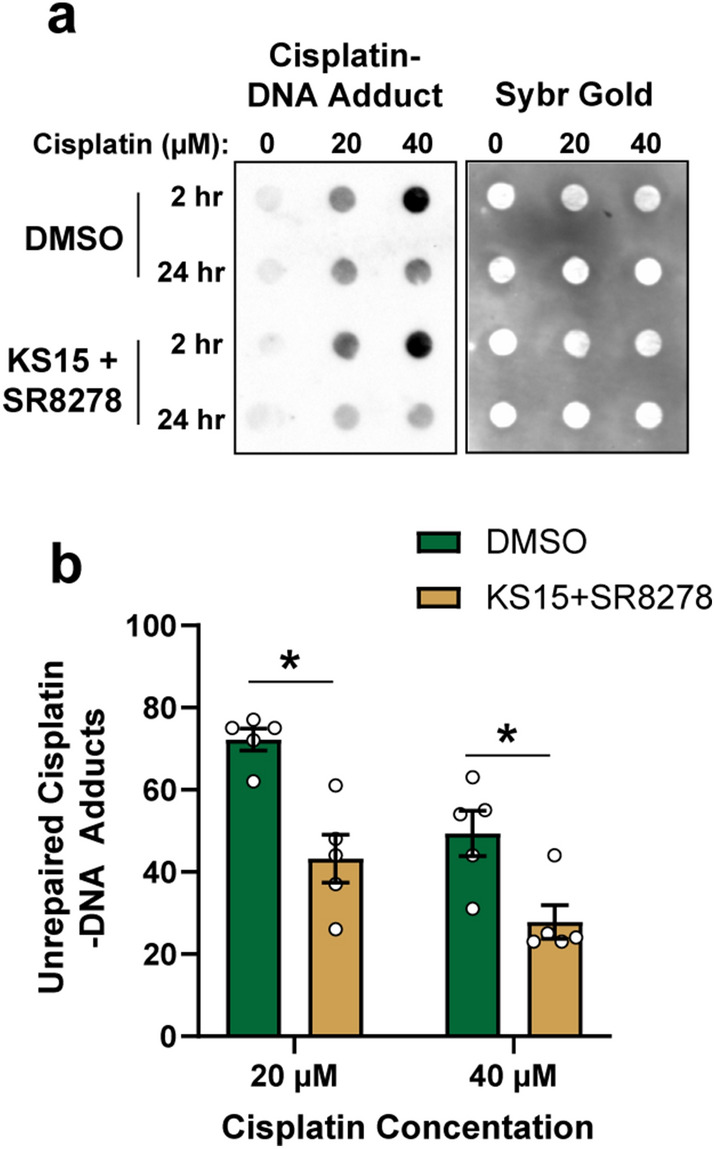
Treatment of cells with KS15 and SR8278 treatment promotes the removal of cisplatin–DNA adducts from genomic DNA. **(a)** U2OS cells were treated with DMSO vehicle or 20 µM KS15 + 10 µM SR8278 along with the indicated concentrations of cisplatin. Genomic DNA was purified from the cells 2 and 24 h later and analyzed by DNA immunoblotting with an anti-cisplatin DNA adduct antibody. Blots were then stained with SybrGold to visualize total DNA. **(b)** Quantitation of relative cisplatin–DNA adduct signal (average and SEM) at 24 h in relative to the 2 h time point (n = 5). The asterisks indicate significant differences in unrepaired cisplatin adducts (t-test, p < 0.05).

XPA is a rate-limiting factor in NER^30^, and thus the increased expression of XPA observed in Fig. 3 may facilitate the removal of cisplatin-induced intra-strand DNA adducts. U2OS cells were treated with vehicle or KS15 and SR8278 in the presence of increasing concentrations of cisplatin, and then genomic DNA was purified for analysis by DNA immunoblotting with an anti-cisplatin–DNA adduct antibody. As shown in Fig. 4A, cisplatin treatment for 2 h induced a dose-dependent induction of adduct formation. Cell culture media was then replaced with fresh medium containing the clock drugs but lacking cisplatin to determine how the clock drugs impact the repair of these DNA adducts after an additional 22 h of incubation. Consistent with XPA’s role in promoting NER and its up-regulation by treatment of cells with KS15 + SR8278, fewer remaining cisplatin–DNA adducts were observed in the cells treated with KS15 and SR8278 at the 24 h time point than in the vehicle-treated cells (Fig. 4A,B).

### KS15 and SR8278 promote G1 cell cycle arrest

**Figure 5:**
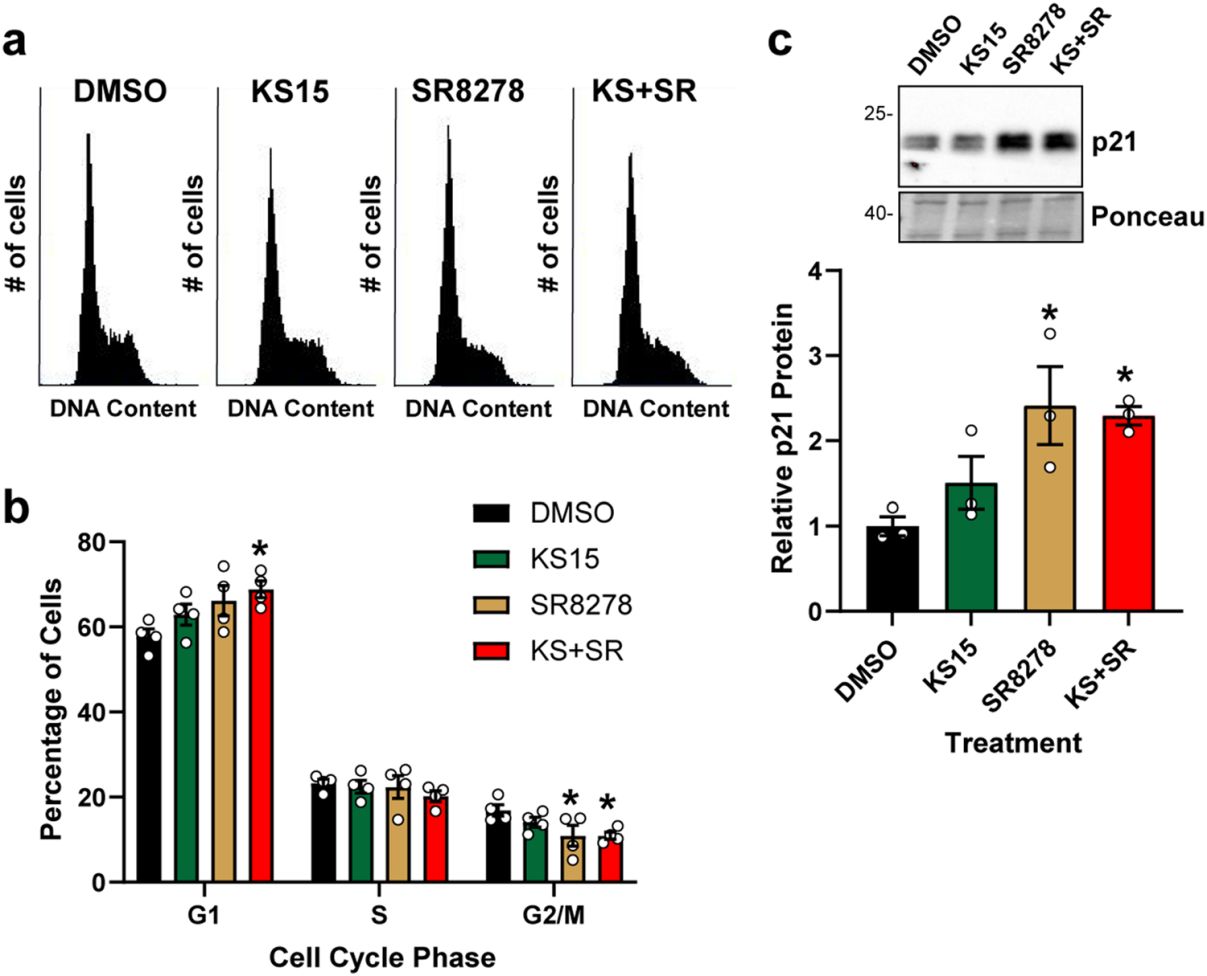
KS15 and SR8278 treatment leads to an increased fraction of cells in G1 phase and to increased expression of p21. **(a)** U2OS cells were treated with DMSO vehicle, 20 µM KS15, 10 µM SR8278, or the combination of KS15 and SR8278 (KS + SR) for 24 h. Cells were fixed, stained with propidium iodide, and analyzed by flow cytometry. Cell cycle distribution from a representative experiment is shown. **(b)** Cells were gated for DNA content to determine the fraction of cells in G1, S, and G2/M phase (average and SEM, n = 3). **(c)** Cell lysates from experiments performed in Fig. 3 were analyzed for p21 protein expression (average and SEM, n = 3). The full-length blot is presented in Supplementary Figure S1. The asterisks indicate significant differences in cell cycle distribution and p21 expression (p < 0.05; one-way ANOVA).

Because the Wee1 kinase prevents the entry of cells into mitosis, the increased expression of Wee1 observed in Fig. 3 may be expected to lead to an enrichment of cells in the G2 phase of the cell cycle. However, when U2OS cells were treated with KS15 and SR8278 (alone and in combination) and analyzed for DNA content by flow cytometry, a modest reduction in cells in late S/2 phase was observed (Fig. 5A). Rather, quantitation of cell cycle distribution from 3 independent experiments showed there to a modest but statistically significant increase in cells in the G1 phase of the cell cycle (Fig. 5B) when cells were treated with the combination of KS15 and SR8278. To identify a potential mechanism for this increased fraction of cells in G1 phase, expression of the cyclin-dependent kinase inhibitory protein p21^31^, which was previously reported to be a target of the circadian clock machinery^32^, was examined. Western blotting showed that p21 protein levels were increased by treatment of SR8278 alone or in combination with KS15 (Fig. 5C). Thus, cell cycle progression is impacted by treatment with small molecules that target circadian clock proteins in a similar manner as for DNA repair.

## Discussion

Though there is growing interest in using small molecule drugs that target the circadian clock to ameliorate the pathophysiology of metabolic disorder and cancer^20,21^, little work has been done to study how these compounds can be used to modulate responses to DNA damage caused by cisplatin and other related agents. The current study is the first to analyze how the CRY inhibitor KS15 and REV-ERB antagonist SR8278 affect the anti-proliferative effects of cisplatin. Because CRY and REV-ERB function to suppress CLOCK-BMAL1 expression and/or activity^8^, pharmacological inhibition of CRY and REV-ERB is expected to de-repress the transcription complex and promote the expression of CCGs that may be relevant for cellular responses to cisplatin. Consistent with this hypothesis, the NER factor XPA and cell cycle regulators Wee1 and p21 were found here to be increased by treatment with KS15 and SR8278 (Figs. 3, 5). This increased gene expression was correlated with more efficient NER (Fig. 4), a higher fraction of cells in G1 phase of the cell cycle (Fig. 5), and resistance to the anti-proliferative effects of cisplatin (Figs. 1, 2 and Table 1).

The work here focused on a limited set of gene products relevant to cisplatin responses, and thus it is possible that additional genes and signaling pathways also contribute to the effects of KS15 and SR8278 on cellular responses to cisplatin. Thus, additional work may be needed to fully understand the effects of these compounds and to determine whether the effects are dependent on the circadian clock machinery. Moreover, as methods for synchronizing the circadian behavior of the cultured cells, such as high serum concentrations or dexamethasone, were not used in this work, it is possible that the effectiveness of KS15 and SR8278 in protecting against cisplatin may be altered by the timing of treatment during normal circadian rhythms. Such approaches in cultured cells in vitro and in animal models in vivo are therefore warranted to advance the idea of using clock modulating compounds to alter cellular responses to DNA damage.

Based on the results of this study, the use of KS15 and SR8278 may be relevant to limit the toxicity caused by cisplatin in normal body tissues such as the kidney and brain, which are known to be impacted by cisplatin chemotherapy regimens. However, these compounds may also limit the effectiveness of cisplatin towards cancer cells. Thus, targeted drug delivery could be necessary to optimize desired therapeutic outcomes. Nonetheless, while the work here focused on compounds that target circadian clock components that are expected to enhance CLOCK-BMAL1 transcriptional output, there are other clock-modulating compounds^21^, including the CRY stabilizer KL001^33^ and REV-ERB agonist SR9009^34^, that in principle may sensitize cells to cisplatin by inhibiting the expression of CCGs important in DNA repair and cisplatin responses. A recent study showed that the combined treatment of glioblastoma stem cells with CRY and REV-ERB agonists in the absence of any DNA damaging agent slowed the growth of the cells in culture in vitro and as tumors in mice in vivo^35^. Future studies should therefore explore these additional classes of clock modulating drugs in the context of cisplatin treatment.

## Materials and methods

### Cell culture and materials

U2OS and HaCaT cells were obtained from ATCC and maintained in DMEM (HyClone SH30243) containing an additional 2 mM l-glutamine (Gibco 205030-081), 100 units/ml penicillin and 100 µg/ml streptomycin (Gibco 15140), and 10% fetal bovine serum (Hyclone SH30109). A549 cells were cultured in F-12 K medium (Gibco 21127-022) containing 10% fetal bovine serum and antibiotics. All cells were grown in a humidified 37 °C incubator with 5% CO_2_. The clock modulating compounds KS15 (Glixx Laboratories GLXC-20604) and SR8278 (Sigma S9576) were prepared as 10 mM stocks in DMSO (Fisher Chemical). Cisplatin (Sigma P4394) was prepared as a 3 mM stock in PBS (HyClone SH30256). Drugs were used at the final concentrations described in the figure legends.

### Cell proliferation assays

Cells were seeded in 96-well plates at a density of 3000–5000 cells per well in 100 µl of media and grown for 2–3 days until confluent. Cells were then treated for 48 h in the presence or absence of the indicated concentrations of KS15, SR8278, and cisplatin. Media was then aspirated, and cells were incubated for either 4 h (U2OS) or 1 h (HaCaT, A549) with 100 µl of culture medium containing 0.25 mg/ml methyl-thiazolyl diphenyl-tetrazolium bromide (MTT; Sigma M5655). After aspiration of the culture media, MTT crystals were solubilized in 100 µl of DMSO (Fisher D128) and absorbance was measured at 570 nm using a Synergy H1 spectrophotometer (Bio-Tek). GraphPad Prism (version 9.0) was used to normalize the absorbance values, perform non-linear regressions, and calculate IC_50_ values.

### mRNA expression analyses

RNA was purified using an RNeasy Plus Micro Kit (Qiagen 74034), quantified on a NanoDrop One spectrophotomer (ThermoFisher), and then reverse transcribed using a QuantiTect Reverse Transcription Kit (Qiagen 205313). Quantitative PCR reactions were prepared using 2× TaqMan Fast Universal PCR Master Mix (ThermoFisher 4352042) and TaqMan probes targeting XPA (Hs00902270), Wee1 (Hs01119384), and beta-2-microglobulin (B2M) (Hs0187842) (Applied Biosystems). DNA was amplified on a CFX96 Real-Time PCR Detection System (Bio-Rad) using an initial 3 min melting step at 95 °C followed by 40 cycles of 95 °C for 10 s and 55 °C for 30 s. The ΔΔC_t_ method was used to calculate fold changes in gene expression using B2M as an internal housekeeping gene.

### Protein immunoblotting

Cells were lysed in cold Triton X-100 lysis buffer (20 mM Tris-HCl, pH 7.5, 150 mM NaCl, 1 mM EDTA, 1 mM EGTA, and 1% Triton X-100) and incubated on ice for 15–20 min with occasional vortexing. Soluble lysates were obtained by centrifugation at 16,000 × *g* in a microcentrifuge (Eppendorf 5415). Equal amounts of protein were separated on either pre-cast 4–20% gradient (Bio-Rad) or home-made 8% Tris–Glycine SDS gels at 200 V using a Bio-Rad Mini-PROTEAN Tetra system. Proteins were transferred to a 0.45 µm nitrocellulose membrane (Cytiva Amersham Protran 45-004-016) at 25 V for 12 min using a Bio-Rad Trans-Blot Turbo semi-dry transfer apparatus and then stained with 0.5% Ponceau S (Sigma P3504) to ensure equal loading and transfer. The blots were blocked in 5% non-fat milk (Kroger) in TBST (Tris-buffered saline containing 0.1% Tween-20) and then probed overnight with primary antibodies against XPA (1:1000 dilution; sc-28353), Wee1 (1:1000; sc-5285), or p21 (1:1000; sc-2646), which were obtained from Santa Cruz Biotechnology. After washing with TBST, blots were probed with 1:5000 dilutions of HRP-coupled anti-mouse or anti-rabbit IgG (Invitrogen 31460 and 31436) secondary antibodies for one hour at room temperature. Chemiluminescence was visualized with Clarity Western ECL substrate (Bio-Rad) or SuperSignal West Femto substrate (Thermo Scientific) using a Molecular Imager Chemi-Doc XRS + imaging system (Bio-Rad). Image Lab (Bio-Rad) was used to quantify signal intensities, which were normalized to the Ponceau S stain.

### DNA immunoblotting

Genomic DNA was purified using a GenElute Mammalian Genomic DNA Miniprep Kit (Sigma G1N350) and quantified using either a NanoDrop One spectrophotometer or Qubit 4.0 fluorometer (Thermo Fisher). Equal amounts of genomic DNA (50–100 ng) were boiled for 10 min, neutralized on ice with an equal volume of 2 M cold ammonium acetate (pH 7.0; Fisher BP326) and then loaded onto a nitrocellulose membrane using a 96-well dot blot apparatus (Bethesda Research Laboratory 1050MM or Bio-Rad Bio-Dot). After drying for 30 min at 80 °C, blots were blocked in milk and probed with anti-cisplatin-modified DNA antibody (1:10,000 dilution; Abcam ab103261) and then HRP-coupled anti-rat IgG secondary antibody (Abcam ab6734). Chemiluminescence was detected and quantified as described for protein immunoblotting. In some cases, blots were stained with SYBR Gold stain (1:10,000; Invitrogen).

### Flow cytometry

Cells were harvested by trypsinization and centrifuged at 1500 rpm for 5 min. After washing with PBS, cells were fixed overnight with 70% ice-cold ethanol at − 20 °C. Fixed cells were washed with PBS, centrifuged at 4000 rpm for 5 min, and resuspended in PBS containing 10 µg/ml RNase A (Fisher BP2539) and 50 µg/ml propidium iodide (Sigma P1470). Cells were analyzed for DNA content using an Accuri C6 flow cytometer. Cell cycle distribution was determined after appropriate gating of cell populations.

### Statistical analyses

All experiments were performed at least three times, and samples sizes are indicated in the figure legends. Differences between treatment groups were evaluated using GraphPad Prism (version 9.0) and either t-tests or one-way ANOVAs. Data were considered statistically significant at p-values less than 0.05. In most graphs, average and SEM are displayed along with values for individual experimental samples.

## Supplementary Information

Supplementary Figure S1.

## Data Availability

All data generated or analysed during this study are included in this published article.
