# Supplementary material for: Pharmacological inhibition of cryptochrome and REV-ERB promotes DNA repair and cell cycle arrest in cisplatin-treated human cells
Source: Sci Rep. 2021 Sep 9;11:17997. doi: 10.1038/s41598-021-97603-x (PMC8429417; doi:10.1038/s41598-021-97603-x)
Supplement: Supplementary file 1 — Supplementary Figure S1. [file 41598_2021_97603_MOESM1_ESM.pdf]

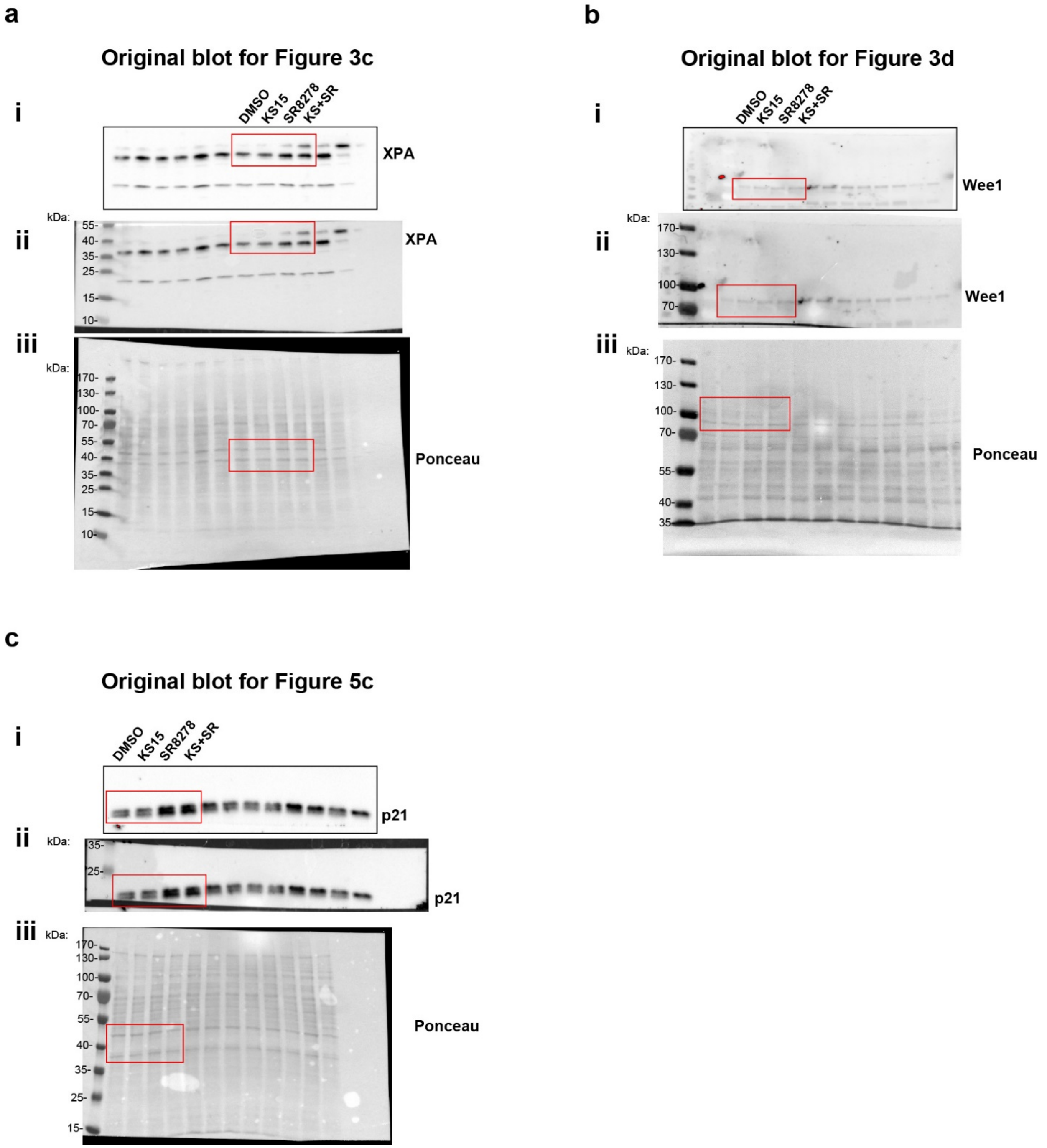

Supplementary Figure S1. Full-length blots for western blot data shown in Figures 3 and 5. For each of the cropped western blots shown in **(a)** Figure 3c (XPA), **(b)** Figure 3d (Wee1), and **(c)** Figure 5c (p21), an original blot showing the non-saturated chemiluminescent signal is shown (i) alone and (ii) merged with a white light image of the blot to show the locations of the molecular weight markers (ii). In addition, the Ponceau-stained blot is provided as a loading control (iii). The red boxes indicate the area that was cropped for the figures shown in the main text of the manuscript. Note that for the Wee1 blot (b), the camera was zoomed in at different levels for the chemiluminescence and white light images, and thus the images do not perfectly align when merged (b ii).
